# The Key Role of Patient Empowerment in the Future Management of Cancer-Related Malnutrition

**DOI:** 10.3390/nu15010235

**Published:** 2023-01-03

**Authors:** Amanda Casirati, Valentina Da Prat, Emanuele Cereda, Francesco Serra, Lorenzo Perrone, Salvatore Corallo, Francesco De Lorenzo, Paolo Pedrazzoli, Riccardo Caccialanza

**Affiliations:** 1 Clinical Nutrition and Dietetics Unit, Fondazione IRCCS Policlinico San Matteo, 27100 Pavia, Italy; 2 Medical Oncology Unit, Fondazione IRCCS Policlinico San Matteo, 27100 Pavia, Italy; 3 Italian Federation of Volunteer-Based Cancer Organizations, 00187 Rome, Italy; 4 Department of Internal Medicine and Medical Therapy, University of Pavia, 27100 Pavia, Italy

Malnutrition is a common condition in cancer patients. It is associated with poor clinical outcomes and higher healthcare costs, with approximately 20–30% of patients dying because of its consequences rather than cancer. The prevalence of cancer-related malnutrition depends on disease stage and localization, ranging from 15% to 40% at diagnosis, and up to 80% in later stages. Patients affected by gastrointestinal tract tumors and advanced-stage disease present the highest rates of malnutrition [1,2].

Nutritional support helps improving clinical outcomes and lowering the risk of mortality in many settings, including patients with head and neck, gastrointestinal, respiratory, and genitourinary cancer. Therefore, all efforts should be made to include nutritional interventions in multimodal oncologic care since diagnosis, even when patients are not severely malnourished [3].

Although it is known that nutritional status impairment affects the efficacy of anticancer treatment and increases the risk of adverse outcomes, cancer-related malnutrition is still under-recognized and under-treated. In fact, the collaboration between oncologists and nutritionists seems to be suboptimal in current clinical practice, as clinical nutrition is often considered as a non-essential part of multimodal cancer care [4].

According to the most recent international guidelines and recommendations [1,5], malnourished patients and patients at risk of malnutrition should be recognized early using nutritional risk screening tools. A comprehensive assessment of nutritional status including anthropometry, body composition, oral intake and inflammatory status evaluation is mandatory to implement an appropriate and timely nutritional support plan. Dietary counseling (DC) is the first-line intervention in malnourished cancer patients. Through the optimization of oral food intake, it acts on mitigating metabolic derangements, maintaining body weight and improving body composition through skeletal muscle mass preservation. DC was demonstrated to improve survival, reduce the risk of modification of planned anticancer treatments, and improve quality of life of cancer patients. If necessary, DC may also include the administration of oral nutritional supplements (ONS). ONS are medical nutrition products used to counteract cancer-related malnutrition when normal food consumption is not sufficient to maintain or increase energy intake. Since ONS have different textures and flavors, they can be easily incorporated into the usual diet and adapted to personal preferences of patients. In case of insufficient energy intake through the oral route, artificial nutrition can be administered through an enteral access (i.e., nasogastric tube, nasojejunal tube, or percutaneous gastrostomy) or through the parenteral route by a central or peripheral venous access [5].

Despite scientific and clinical evidence, many issues remain to be addressed.

For instance, no clear indication is available on how to manage patients with a preserved nutritional status at diagnosis. In this population, there is a high risk of overlooking the development of malnutrition during the illness trajectory. This is more frequent in some cancer types (i.e., gastrointestinal, head and neck and lung cancer), in advanced disease stage and in case of aggressive treatment modalities, which are likely to worsen nutritional status even in non-previously malnourished patients [5]. In case of treatment toxicities, the early identification and management of nutrition impact symptoms (i.e., nausea, vomiting, anorexia, dysphagia, dysgeusia and diarrhea) can increase survival rates, as seen in patients with metastatic esophagogastric cancer [6].

A critical problem is the marked heterogeneity in terms of health care organization and resources. Clinical nutrition (CN) units are often insufficient or unevenly distributed across the country, and most oncology units do not have dedicated dietitians [4]. In this scenario, the patient’s involvement is of crucial importance for improving the suitability of nutritional interventions. Patients should be aware that cancer-induced systemic inflammation might affect nutrient intake and metabolism, leading to weight loss and muscle mass reduction. Therefore, they should be educated on which parameters to monitor at home: body weight, food intake, nutrition impact symptoms, reduced muscle strength, prolonged bed rest, chronic pain, and psychological stress. Patients should be instructed to contact their reference CN unit as soon as weight loss is detected, food intake is significantly reduced or symptoms occur to discuss possible interventions. According to disease type and treatment plan, it may be useful to perform an anticipatory dietary counseling (DC) session in order to make patients aware of all the possible symptoms and side effects that may worsen their nutritional status. The aim of DC is not only to optimize food intake and to educate patients on how to satisfy energy–protein requirements with high-calorie and high-protein food, but also to advice against unproven diets [1].

Nutrition also has a deep psychological value. Patients often consider food as a cure to reduce treatment side effects and organ toxicity, and to protect and stimulate the immune system. DC should provide support, discuss realistic expectations, and explore the benefits and risks of diets in an educational, interactive process aimed at increasing patients’ knowledge about their health status and to positively influence attitudes and behavior. When developing effective self-care skills, patients will be able to make appropriate choices to maintain optimal health.

The concept of “empowerment” refers to the intention to promote patients’ abilities of taking greater control of their own health in the context of safety promotion. It is essential that clinical nutritionists understand patients’ perspectives, with a receptive attitude towards their fears and priorities. Poor communication may produce a rupture in the therapeutic relationship, increasing patients’ risk of approaching harmful and useless diets that increase malnutrition risk [7].

Inadequate nutritional management of cancer patients is not ethically acceptable, as nutritional support contributes to enhance the efficacy of anticancer therapies and to ameliorate survival outcomes and quality of life by counteracting malnutrition. DC has a primary role not only in limiting the detrimental impact of cancer on nutritional status, but also in helping patients to develop their capacities of asserting control over their health and managing the psychological aspects of dietary modifications. Through an adequate educational process that provides both information based on scientific evidence and effective communication, it is possible to empower cancer patients as active and autonomous participants in the nutritional care process. This will facilitate the improvement and dissemination of appropriate and timely nutritional support, which will provide relevant clinical and economic advantages to the whole health care system.

## Data Availability

Not applicable.
